# A neural network model combining the successor representation and actor-critic methods reveals effective biological use of the representation

**DOI:** 10.3389/fncom.2025.1647462

**Published:** 2025-11-26

**Authors:** Takayuki Tsurumi, Kenji Morita

**Affiliations:** 1 Physical and Health Education, Graduate School of Education, The University of Tokyo, Tokyo, Japan; 2 International Research Center for Neurointelligence (WPI-IRCN), The University of Tokyo, Tokyo, Japan

**Keywords:** successor representation, actor-critic, neural network, reinforcement learning, striatum

## Abstract

In learning goal-directed behavior, state representation is important for adapting to the environment and achieving goals. A predictive state representation called successive representation (SR) has recently attracted attention as a candidate for state representation in animal brains, especially in the hippocampus. The relationship between the SR and the animal brain has been studied, and several neural network models for computing the SR have been proposed based on the findings. However, studies on implementation of the SR involving action selection have not yet advanced significantly. Therefore, we explore possible mechanisms by which the SR is utilized biologically for action selection and learning optimal action policies. The actor-critic architecture is a promising model of animal behavioral learning in terms of its correspondence to the anatomy and function of the basal ganglia, so it is suitable for our purpose. In this study, we construct neural network models for behavioral learning using the SR. By using them to perform reinforcement learning, we investigate their properties. Specifically, we investigated the effect of using different state representations for the actor and critic in the actor-critic method, and also compared the actor-critic method with Q-learning and SARSA. We found the difference between the effect of using the SR for the actor and the effect of using the SR for the critic in the actor-critic method, and observed that using the SR in conjunction with one-hot encoding makes it possible to learn with the benefits of both representations. These results suggest the possibility that the striatum can learn using multiple state representations complementarily.

## 1 Introduction

In learning goal-directed behavior, state representation is important for adapting to the environment and achieving goals.

The successor representation (SR) (Dayan, 1993, 2002) has recently attracted attention as a candidate for state representation in the animal brain, especially the hippocampus. The SR is a state representation based on the prediction of state transitions. Links between the SR and the animal brain have been studied. For example, it is said that assuming that animals use SR-like state representations explains the results of Tolman's experiments on latent learning (Tolman, 1948; Russek et al., 2017). In addition, some of the properties of hippocampal place cell activity are common to the SR (Stachenfeld et al., 2017). Given this background, several neural network models for computing the SR have been proposed (Burton et al., 2023; Fang et al., 2023; George et al., 2023). One of them (Fang et al., 2023) is a model that uses a recurrent neural network (RNN) in which the SR emerges as a result of the RNN's dynamics and the plasticity of its coupling weights. Another study (Burton et al., 2023) shows the mathematical equivalence of TD(λ) learning with the SR and weight update of a spiking neural network (SNN) derived from inputs assuming hippocampal place cells and spike-timing-dependent plasticity (STDP). Another model (George et al., 2023) also uses STDP. All those models are considered for implementation in the hippocampus.

The question of how information about the external world is represented in the brain should be considered simultaneously with the question of how the brain uses it to make behavioral choices. This is because all variables related to the brain can be said to represent information from the outside world in the sense that they are influenced by sensory input, and therefore, what matters is how they are reflected in behavioral output. However, the previous studies (Burton et al., 2023; Fang et al., 2023; George et al., 2023) those proposed models for computing the SR did not contain experiments on ways of utilizing the SR in the brain for action selection.

Thus, while several studies on the biological implementation of the SR have emerged, studies on implementation involving action selection have not yet advanced significantly. Therefore, we explore possible mechanisms by which the SR is utilized biologically for action selection and learning optimal action policies.

The actor-critic method (Barto, 1995; Houk et al., 1995) is a reinforcement learning method often used as a model of behavioral learning in animals. It was originally devised based on physiological and anatomical findings of the basal ganglia (Houk et al., 1995). To this day, the actor-critic method is often used as a model for learning by the basal ganglia (Khamassi et al., 2005; Dunovan and Verstynen, 2016). It is hypothesized that the dorsolateral striatum corresponds to the Actor and the ventral striatum to the Critic (Takahashi et al., 2008). The actor-critic method consists of an “actor” that determines actions and a “critic” that evaluates those actions. The actor learns policies while the critic learns value functions, and state representations such as the SR can be used in the learning. It is also possible to use different state representations for the actor and critic. This corresponds to the use of different state representations in the dorsolateral striatum and the ventral striatum, according to the hypothesis regarding the striatum mentioned above. By conducting simulations where the actor and critic employ different state representations, we expect to gain insights into the utilization of multiple state representations in biological systems.

The actor-critic method is not the only model for behavioral learning in animals. While the actor-critic method learns value and policy separately, there are also models that learn the state-action value function representing the value of actions and determine actions based on it. Q-learning (Watkins and Dayan, 1992) and SARSA (Rummery and Niranjan, 1994) are representative methods for learning the state-action value function. It has been suggested that dopamine neurons in the ventral tegmental area (VTA) and the substantia nigra pars compacta (SNc) encode RPE for Q-learning (Roesch et al., 2007) and SARSA (Morris et al., 2006), respectively. SR can also be used to learn state-action value functions (Russek et al., 2017). In this case, SR based on transition probabilities between state-action pairs is employed.

In this study, we construct neural network models for action selection using state representations including the SR. By using the models to perform reinforcement learning, we investigate their properties. Specifically, we examine in detail the differences when using the SR for the actor, critic, or both in the actor-critic method. We also examine SARSA and Q-learning using the SR. Through these investigations, we explore possible mechanisms by which the SR is utilized biologically for action selection and learning optimal action policies.

## 2 Materials and methods

### 2.1 The successor representation

The goal of reinforcement learning is to maximize the value function:


$$\begin{array}{l} V^{\pi }\left(s\right)=E_{\pi }\left[\overset{\infty }{\underset{t=0}{\sum }}\gamma^{t}r_{t}|s_{0}=s\right]. \end{array}$$ (1)

Here, *r*_*t*_ is the reward given at time *t*, *s*_*t*_ is the state at time *t*, and γ is a parameter called discount factor. 𝔼_π_[·] means expected values when the agent acts according to a policy π. The value function represents the expected cumulative discounted reward.

The value function can be approximated with some basis functions as follows:


$$\begin{array}{l} V\left(s\right)=w^{⊤}x\left(s\right). \end{array}$$ (2)

Here, *V* is the estimated value function, ***w*** is a weight vector, and ***x***(*s*) is a feature vector representing state *s*. The weight ***w*** can be learned by standard TD learning adapted for linear function approximation:


$$\begin{array}{l} w\leftarrow w+\alpha^{w}\delta x\left(s\right). \end{array}$$ (3)

where α^*w*^ is a learning rate and δ is the TD error. The TD error is defined as


$$\begin{array}{l} \delta =r_{t}+\gamma V\left(s_{t+1}\right)-V\left(s_{t}\right). \end{array}$$ (4)

The successor representation (SR) is a state representation based on the prediction of state transitions. The SR matrix *M* is defined as


$$\begin{array}{l} M_{s,s^{\prime }}=\overset{\infty }{\underset{t=0}{\sum }}\gamma^{t}P\left(s_{t}=s^{\prime }|s_{0}=s\right). \end{array}$$ (5)

The rows of the SR matrix can be used as ***x***(*s*) in Equation 2 (Russek et al., 2017):


$$\begin{array}{l} V\left(s\right)=\sum_{s^{\prime }}M_{s,s^{\prime }}w_{s^{\prime }} \end{array}$$ (6)

where $w_{s^{\prime }}$ is a component of vector ***w*** corresponding to a state *s*′. Then, according to Equation 3, ***w*** can be learned by


$$\begin{array}{l} w_{s^{\prime }}\leftarrow w_{s^{\prime }}+\alpha \delta M_{s_{t},s^{\prime }} \end{array}$$ (7)

for all states *s*′.

### 2.2 Learning the successor representation

We use one of the methods of learning SR using neural networks proposed in a previous study (Fang et al., 2023).

A recurrent neural network (RNN) is used to compute the SR. It is assumed that the transition probability matrix *T* is encoded in the synaptic weights of the RNN. Then, the steady-state activity of the network in response to one-hot input ***ϕ*** retrieves a row of the SR matrix, *M*^⊤^***ϕ***.

The dynamics of the RNN is defined with the following equation:


$$\begin{array}{l} x\left(t+1\right)=\gamma Jf\left(x\left(t\right)\right)+\phi \left(t\right). \end{array}$$ (8)

Here, ***x*** is the activity of RNN neurons, *J* is the weight matrix of RNN, *f* is an activation function, ϕ is the input, and γ is a scaling factor of recurrent activation. This dynamics leads to the steady-state activity


$$\begin{array}{l} x_{\text{ss}}={\left(I-\gamma J\right)}^{-1}\phi , \end{array}$$ (9)

when *f* is the identity function.

The weight *J* is updated as follows:


$$\begin{array}{l} J\leftarrow J+\eta x\left(t\right)x{\left(t-1\right)}^{⊤}-\eta Jx\left(t-1\right)x{\left(t-1\right)}^{⊤} \end{array}$$ (10)

where η is a learning rate. For each synapse,


$$\begin{array}{l} J_{ij}\leftarrow J_{ij}+\eta x_{i}\left(t\right)x_{j}\left(t-1\right)-\eta x_{j}\left(t-1\right)\underset{k}{\sum }J_{ik}x_{k}\left(t-1\right). \end{array}$$ (11)

The first term is a temporally asymmetric potentiation term which is similar to spike-timing-dependent plasticity (STDP). The second term is a form of synaptic depotentiation, and similar inhibitory effects are known to be elements of hippocampal learning (Kullmann and Lamsa, 2007; Lamsa et al., 2007).

Although it works with a static learning rate, to accelerate learning, the authors introduced an adaptive learning rate calculated by $n\left(t\right)=\underset{t^{\prime }<t}{\sum }x\left(t^{\prime }\right),\eta =\min\left(\frac{1}{n_{j}\left(t\right)},1\right)$ for synapses from neuron *j*. Modulating synaptic learning rates as a function of neural activity is consistent with experimental observations of metaplasticity (Abraham and Bear, 1996; Abraham, 2008; Hulme et al., 2014).

The authors assumed that the timescale of the plasticity is longer than the timescale of the RNN dynamics and that ***x*** can be regarded as converging to the steady state in the update of the weight. Under this assumption, the plasticity (Equation 10) leads to


$$\begin{array}{l} J=T^{⊤} \end{array}$$ (12)

where *T* is the transition probability matrix. *T* gives the probability that the agent transitions from a state *s* to a state *s*′ in one time step: $T_{s,s^{\prime }}=P\left(s_{t+1}=s^{\prime }|s_{t}=s\right)$. From Equations 9, 12 and


$$\begin{array}{l} M=\overset{\infty }{\underset{t=0}{\sum }}\gamma^{t}T^{t}={\left(I-\gamma T\right)}^{-1}, \end{array}$$ (13)

which is derived from Equation 5, we obtain


$$\begin{array}{l} x_{\text{ss}}=M^{⊤}\phi \end{array}$$ (14)

When *f* is a hyperbolic tangent, the steady state approximates the rows of the SR matrix, and the model becomes stable for larger γ values compared to when *f* is the identity function (Fang et al., 2023). Therefore, we use tanh as *f*. As in the previous study, we use ***x*** after repeating the update (Equation 8) for *t*_max_ steps such that $\gamma_{\text{max}}^{t}<10^{-4}$ as the steady-state activity ***x***_ss_.

### 2.3 Actor-critic

We adopt an actor-critic method with a policy gradient method (Sutton and Barto, 2018).

The value function is approximated by


$$\begin{array}{l} V\left(s\right)=w^{⊤}x\left(s\right) \end{array}$$ (15)

and the weight ***w*** is learned by


$$\begin{array}{l} w\leftarrow w+\alpha^{w}\delta x\left(s\right), \end{array}$$ (16)

as described in Section 2.1. The inner product in Equation 15 is calculated by synapses whose weight represents ***w*** and activity of presynaptic cells represents ***x***(*s*). The weight update Equation 16 can be interpreted as synaptic plasticity dependent on presynaptic cell activity.

A policy π is defined with an exponential soft-max distribution:


$$\begin{array}{l} \pi \left(a|s,\theta \right)=\frac{e^{\beta h\left(s,a,\theta \right)}}{\sum_{b}e^{\beta h\left(s,b,\theta \right)}} \end{array}$$ (17)

where π(*a*|*s*, ***θ***) represents the probability of choosing action *a* in state *s* parametrized by ***θ***, *h*(*s, a*, ***θ***) represents preference of action *a* in state *s* parametrized by ***θ***, and β is a parameter scaling the preference. For the tasks we use, which are maze tasks with 4 actions, preference *h* is defined as


$$\begin{array}{l} h\left(s,a_{i},\theta \right)=\theta_{i}^{⊤}x\left(s\right)\;\left(i=1,2,3,4\right), \end{array}$$ (18)

where ***x***(*s*) is the feature vector representing state *s* and


$$\begin{array}{l} \theta =\left(\begin{array}{c} \theta_{1}\\ \theta_{2}\\ \theta_{3}\\ \theta_{4} \end{array}\right). \end{array}$$ (19)

The parameter *θ* is learned by a learning rule of policy gradient methods (Sutton and Barto, 2018):


$$\begin{array}{l} \theta \leftarrow \theta +\alpha^{\theta }\gamma^{t}\delta \nabla_{\theta }\ln\pi \left(a|s,\theta \right) \end{array}$$ (20)

when the agent chose action *a* at state *s*, where α_θ_ is a learning rate and γ is a discount factor. For policies expressed by Equation 17, this becomes


$$\begin{array}{l} \theta_{k}\leftarrow \theta_{k}+\alpha^{\theta }\gamma^{t}\delta \left(\delta_{ik}-\pi \left(a_{k}|s,\theta \right)\right)x\left(s\right)\;\left(k=1,2,3,4\right) \end{array}$$ (21)

when the agent chose action *a*_*i*_ at state *s*. Here, δ_*ik*_ is Kronecker delta. We omit γ^*t*^ for learning efficiency. The inner product in Equation 18 is calculated by synapses whose weight represents ***θ*** and activity of presynaptic cells represents ***x***(*s*). The learning rule Equation 21 can be interpreted as synaptic plasticity dependent on presynaptic and postsynaptic cell activity.

We use the row corresponding to state *s* of the SR matrix or the one-hot vector corresponding to state *s* as the feature vector ***x***(*s*) in Equations 15, 18. The row corresponding to state *s* of the SR matrix is obtained as the steady-state activity ***x***_ss_ of the RNN in Equation 14 by making ***ϕ*** the one-hot vector corresponding to state *s*.

**Figure 1:**
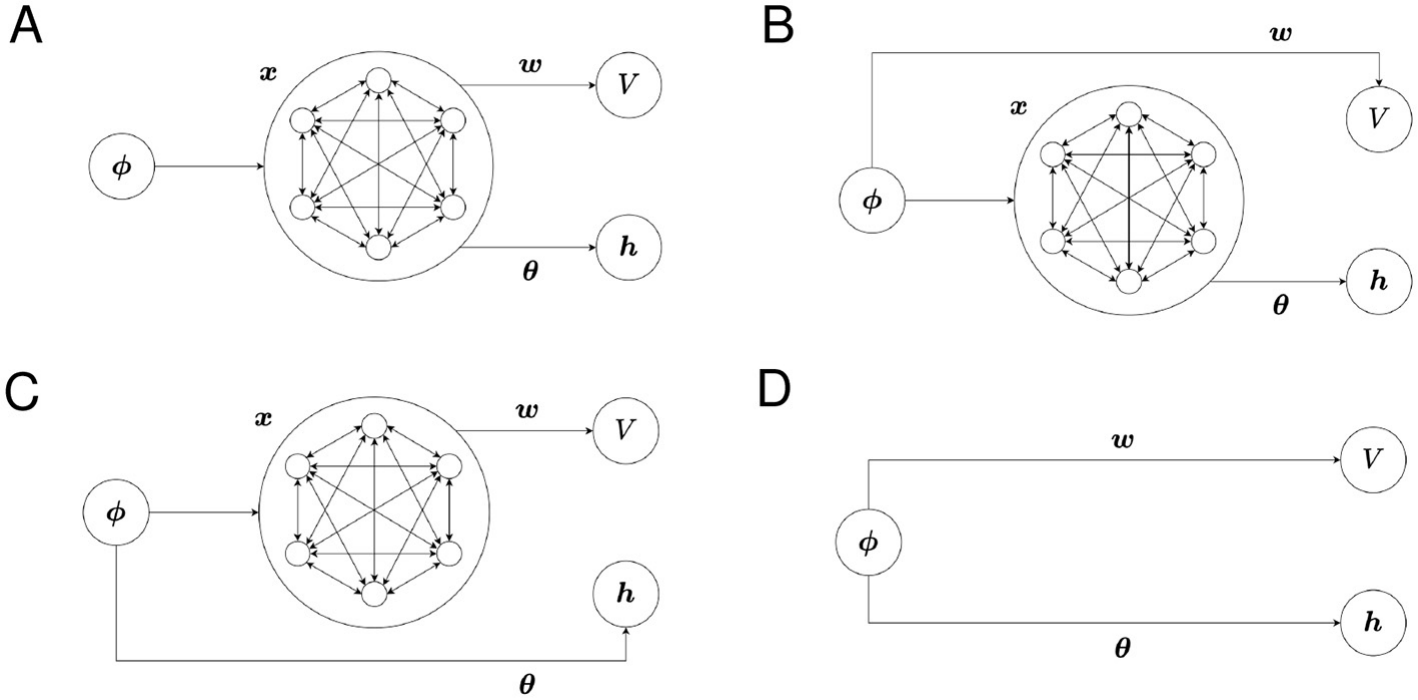
The structure of the proposed model for each combination of state representations. **(A)** Critic: SR, Actor: SR, **(B)** Critic: one-hot, Actor: SR, **(C)** Critic: SR, Actor: one-hot, and **(D)** Critic: one-hot, Actor: one-hot.

We refer to the use of ***x*** in Equation 15 as “using ***x*** for the Critic” and the use of ***x*** in Equation 18 as “using ***x*** for the Actor.” Different feature vectors can be used for the Critic and the Actor. The structures of our model with possible combinations of state representations are shown in Figures 1A–D.

### 2.4 Q-learning and SARSA

We adopt Q-learning and SARSA as representative methods for learning the state-action value function. The state-action value function is defined by


$$\begin{array}{l} Q^{\pi }\left(s,a\right)=E_{\pi }\left[\overset{\infty }{\underset{t=0}{\sum }}\gamma^{t}r_{t}|s_{0}=s,a_{0}=a\right]. \end{array}$$ (22)

The state-action value function can be approximated with some basis functions as follows:


$$\begin{array}{l} Q\left(s,a\right)=w^{⊤}x\left(sa\right). \end{array}$$ (23)

Here, *Q* is the estimated state-action value function, ***w*** is a weight vector, and ***x***(*sa*) is a feature vector representing state-action pair *sa*. The update of ***w*** in Q-learning adapted for linear function approximation is


$$\begin{array}{l} w\leftarrow w+\alpha^{w}\delta x\left(sa\right) \end{array}$$ (24)

where


$$\begin{array}{l} \delta =r_{t}+\gamma \underset{a}{\max}Q\left(s_{t+1},a\right)-Q\left(s_{t},a_{t}\right). \end{array}$$ (25)

The update of ***w*** in SARSA adapted for linear function approximation is


$$\begin{array}{l} w\leftarrow w+\alpha^{w}\delta x\left(sa\right) \end{array}$$ (26)

where


$$\begin{array}{l} \delta =r_{t}+\gamma Q\left(s_{t+1},a_{t+1}\right)-Q\left(s_{t},a_{t}\right). \end{array}$$ (27)

A state-action version of the successor representation matrix is defined as


$$\begin{array}{l} H_{sa,s^{\prime }a^{\prime }}=\overset{\infty }{\underset{t=0}{\sum }}\gamma^{t}P\left(s_{t}=s^{\prime },a_{t}=a^{\prime }|s_{0}=s,a_{0}=a\right). \end{array}$$ (28)

We use the row corresponding to state-action pair *sa* of the SR matrix or the one-hot vector corresponding to state-action pair *sa* as the feature vector ***x***(*sa*) in Equation 23. Using a version of the RNN in Section 2.2 where each neuron corresponds to a state-action pair instead of a state, the row corresponding to state-action pair *sa* of the SR matrix is obtained as the steady-state activity ***x***_ss_ of the RNN by making ***ϕ*** the one-hot vector corresponding to state-action pair *sa*. The inner product in Equation 23 is calculated by synapses whose weight represents ***w*** and activity of presynaptic cells represents ***x***(*sa*).

We use policies similar to Equation 17:


$$\begin{array}{l} \pi \left(a|s,\theta \right)=\frac{e^{\beta Q\left(s,a\right)}}{\sum_{b}e^{\beta Q\left(s,b\right)}}. \end{array}$$ (29)

We calculate the state value function by


$$\begin{array}{l} V\left(s\right)=\underset{a}{\sum }\pi \left(a|s,\theta \right)Q\left(s,a\right) \end{array}$$ (30)

when we visualize it.

### 2.5 Parameters

Unless otherwise noted, the following values were used for each parameter: γ = 0.8, α_*w*_ = 0.3, α_θ_ = 0.3, β = 1. The initial values of ***x***, *J*, ***w***, ***θ*** are a zero vector or a zero matrix.

### 2.6 Tasks

The tasks we used are similar to the latent learning task and the policy revaluation task in a previous study (Russek et al., 2017).

#### 2.6.1 Water maze task

This task is intended to examine the basic performance of the model.

We used a grid world without barriers, which is analogous to water mazes. Each position in the grid world is treated as a single state. There are four actions: moving up, down, left, or right. Actions toward the walls are excluded from the choices. The agent starts from the upper left corner and a reward is placed at the lower right corner. When the agent reaches the goal, one trial ends and it starts again from the upper left corner.

When the SR is used, the agent first learns the state representation without reward. During this, actions are randomly selected. When one-hot encoding is used, the state representation is treated as given. Then, a reward is placed and the agent learns the value function and policy. During this, the state representation is fixed.

#### 2.6.2 Barrier maze task

We use mazes generated by the method described in Section 2.7. Actions toward the barriers are excluded from the choices. Other than that, it is the same as the water maze task.

#### 2.6.3 Policy revaluation task

The SR is said to enable quick adaptation to changes in the environment (Russek et al., 2017). Therefore, in order to see the adaptability of this model to changes in the environment, we conducted an experiment in which the arrangement of rewards was changed in the middle. Punishment (negative reward) is also placed in this task.

We use a specific maze generated by the method described in Section 2.7.

When the SR is used, the agent first learns the state representation without reward. During this, actions are randomly selected. When one-hot encoding is used, the state representation is treated as given. Then, the agent learns value and action in each placement shown in order. The location marked “S” represents the starting point, the red location represents the reward location, and the blue location represents the punishment location. The agent starts from the upper left corner. When the agent reaches the reward or punishment location, it restarts from the starting point. At first, a reward is placed at the lower right corner and a negative reward is placed at the upper right corner. Training is performed for 20 reward trials in this environment. Then, the positions of reward and punishment are reversed. Training is performed for 20 reward trials in the new environment. During this, the state representation is fixed.

### 2.7 Automatic maze generation

We generate barriers on a 7-by-7 grid world. Simply generating barriers randomly can result in the start and the reward location being separated by barriers, making it impossible to reach the reward. Instead of regenerating the maze if the space is divided, we adopt a generation method that avoids division. To clearly demonstrate differences in results of different learning methods and state representations, mazes are constructed by generating narrow, short paths with dead ends. Two paths of each of the lengths 2, 3, and 4 are generated in random order. Path generation is performed by extending a path randomly from a starting point and making an entrance of the path at its endpoint (the starting point becomes a dead end). When generating paths, states adjacent (including diagonally) to existing paths are excluded. First, paths whose starting point of generation is the top-left, top-right, bottom-left, and bottom-right corners are generated in order. After that, starting points of generation are randomly determined. This generation method ensures the space remains undivided.

## 3 Results

### 3.1 Water maze task

**Figure 2:**
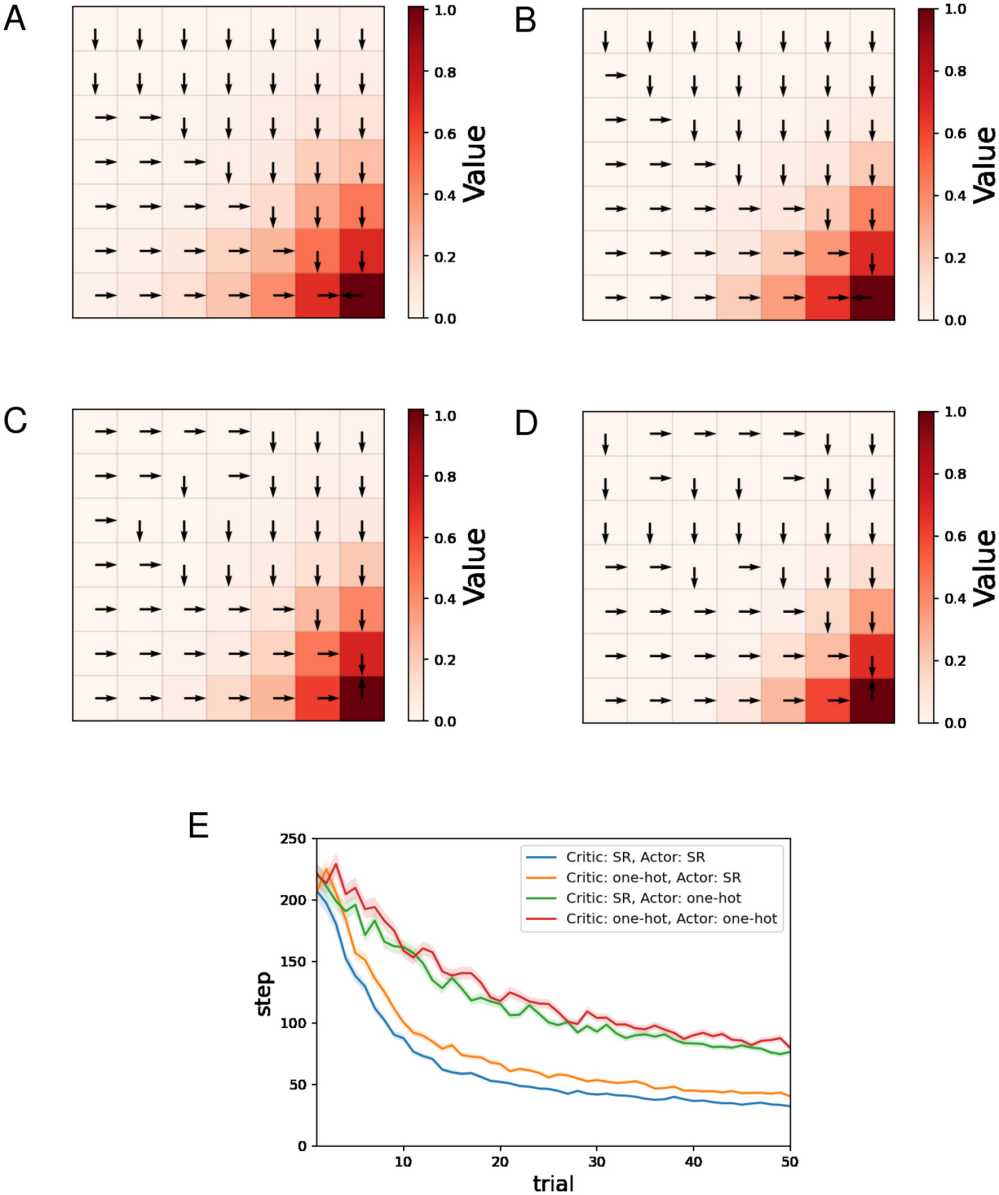
The results of the water maze task regarding the actor-critic method. **(A–D)** Typical examples of the learned value function and optimal actions at the 50th trial. **(A)** Critic: SR, Actor: SR, **(B)** Critic: one-hot, Actor: SR, **(C)** Critic: SR, Actor: one-hot, **(D)** Critic: one-hot, Actor: one-hot, and **(E)** performance comparison between four combinations of state representations.

Figures 2A–D show typical examples of the learned value function and optimal actions. The learned value function is depicted in a color map, and the direction of the action with the highest preference in each state is indicated by an arrow. We can see that learning of the value function proceeds faster when the SR is used for both the Critic and the Actor (Figure 2A) than when one-hot encoding is used for both (Figure 2D). Appropriate policies were learned with all four combinations of state representations.

Figure 2E shows the mean and standard error of the number of steps for each trial while training sessions of 50 trials were performed 500 times. The decreasing trends in the number of steps mean successful learning. The plots in Figure 2E indicate that using the SR for the Actor enhances learning efficiency, while the effect of using the SR for the Critic is small in comparison.

**Figure 3:**
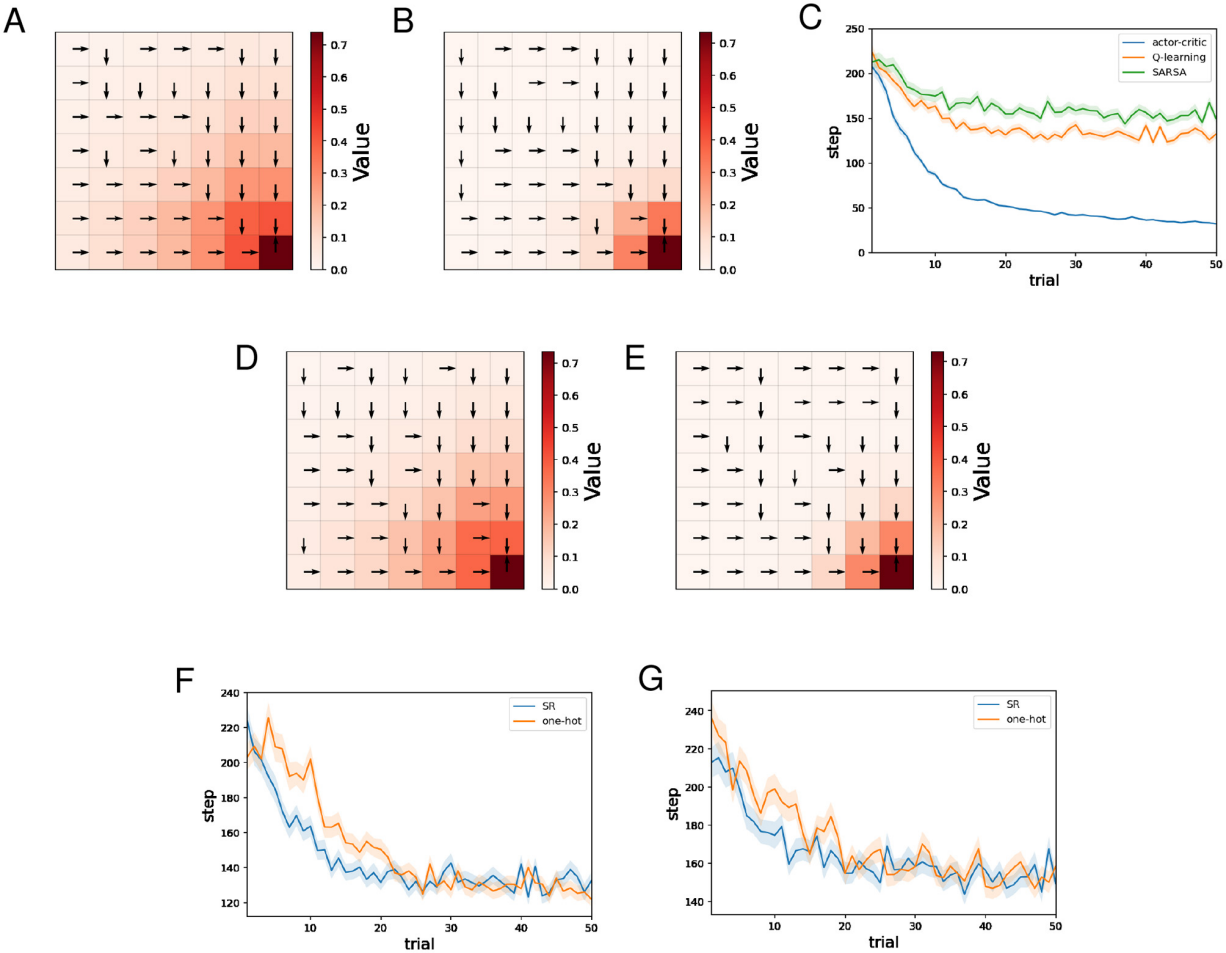
The results of the water maze task regarding Q-learning and SARSA. **(A, B)** Typical examples of the learned value function and optimal actions at the 50th trial of Q-learning **(A)**/SARSA **(B)** using the SR. **(C)** Performance comparison between the actor-critic method, Q-learning, and SARSA. **(D, E)** Typical examples of the learned value function and optimal actions at the 50th trial of Q-learning **(D)**/SARSA **(E)** using one-hot encoding. **(F)** Performance comparison between Q-learning using the SR and Q-learning using one-hot encoding. **(G)** Performance comparison between SARSA using the SR and Q-learning using one-hot encoding.

Figures 3A, B show typical examples of the learned value function and optimal actions for Q-learning and SARSA using the SR, respectively. We can see that learning of the value function proceeds faster for Q-learning than SARSA. This result is natural considering that under the same conditions, an update of *Q*(*s, a*) in Q-learning is larger than or equal to that in SARSA because $\underset{a}{\max}Q\left(s_{t+1},a\right),\geq Q\left(s_{t+1},a_{t+1}\right)$ in Equations 25, 27.

Figure 3C shows the mean and standard error of the number of steps for each trial while training sessions of 50 trials were performed 500 times, for different learning methods using the SR. As for the actor-critic method (hereafter referred to as AC), the SR is used both for the Critic and the Actor. Note that learning of the SR occasionally failed for Q-learning and SARSA, and such sessions are excluded (also for figures below). This phenomenon has been mentioned in the previous study (Fang et al., 2023) that proposed the model for learning SR. The frequency of this failure was less than 10 times out of 500 times. We can see that learning by AC was significantly faster than Q-learning and SARSA, while Q-learning was slightly faster than SARSA. Q-learning and SARSA showed stagnation in learning. Given that both Q-learning and SARSA correctly learned the optimal action at each state, the large number of steps to reach the reward location can be attributed to the small difference between the probability of selecting the optimal action and the probability of selecting other actions. Indeed, in locations distant from the reward location, Q-values are small, and therefore the difference between the Q-value of the optimal action and that of other actions is also expected to be small. In contrast, with AC, the difference between the preference (denoted by *h*) of the optimal action and that of other actions can become sufficiently large as learning progresses, even in locations distant from the reward location. The fact that Q-learning was slightly faster than SARSA is thought to be a consequence of Q-learning learning the value function faster, as mentioned earlier. The slightly faster learning speed of Q-learning compared to SARSA can be interpreted as a consequence of Q-learning learning the value function faster than SARSA.

Figures 3D, E show typical examples of the learned value function and optimal actions for Q-learning and SARSA using one-hot encoding, respectively. In both cases, there is no significant difference compared to when the SR is used.

Figure 3F shows the mean and standard error of the number of steps for each trial while training sessions of 50 trials were performed 500 times, for Q-learning with the SR and Q-learning with one-hot encoding. Learning by Q-learning with the SR was slightly faster than learning by Q-learning with one-hot encoding. Figure 3G shows the mean and standard error of the number of steps for each trial while training sessions of 50 trials were performed 500 times, for SARSA with the SR and SARSA with one-hot encoding. There is no significant difference in learning speed.

### 3.2 Barrier maze task

**Figure 4:**
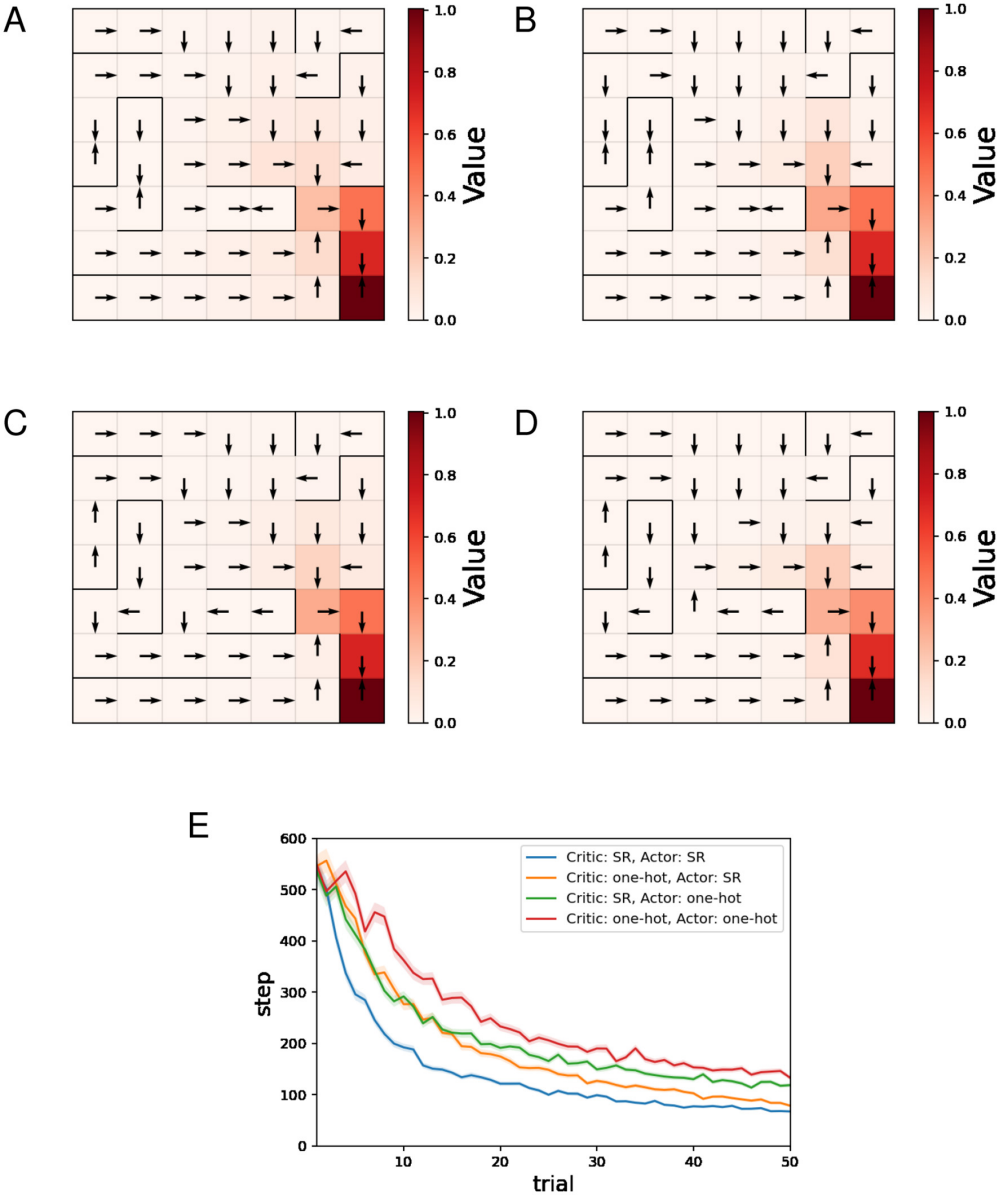
The results of the barrier maze task regarding the actor-critic method. **(A–D)** Typical examples of the learned value function and optimal actions at the 50th trial. **(A)** Critic: SR, Actor: SR, **(B)** Critic: one-hot, Actor: SR, **(C)** Critic: SR, Actor: one-hot, **(D)** Critic: one-hot, Actor: one-hot, and **(E)** performance comparison between 4 combinations of state representations.

Figures 4A–D show typical examples of the learned value function and optimal actions. There is no significant difference in the learned value function across the four combinations of state representations. When the SR is used for the Actor (Figures 4A, B), the learned optimal actions are not appropriate at some locations in short paths with dead ends. The preference for actions in one state is influenced by the learning of actions in other states when the SR is used for the Actor, and this can result in such inappropriate policies.

Figure 4E shows the mean and standard error of the number of steps for each trial while training sessions of 50 trials were performed 500 times. The decreasing trends in the number of steps mean successful learning. The plots in Figure 4E indicate that both using the SR for the Critic and using the SR for the Actor enhance learning efficiency, with using the SR for the Actor having the greater effect.

As these results show, when the SR is used for the Critic and one-hot encoding is used for the Actor, appropriate policies can be learned and learning is faster than when one-hot encoding is used for both, making it a promising combination of state representations.

**Figure 5:**
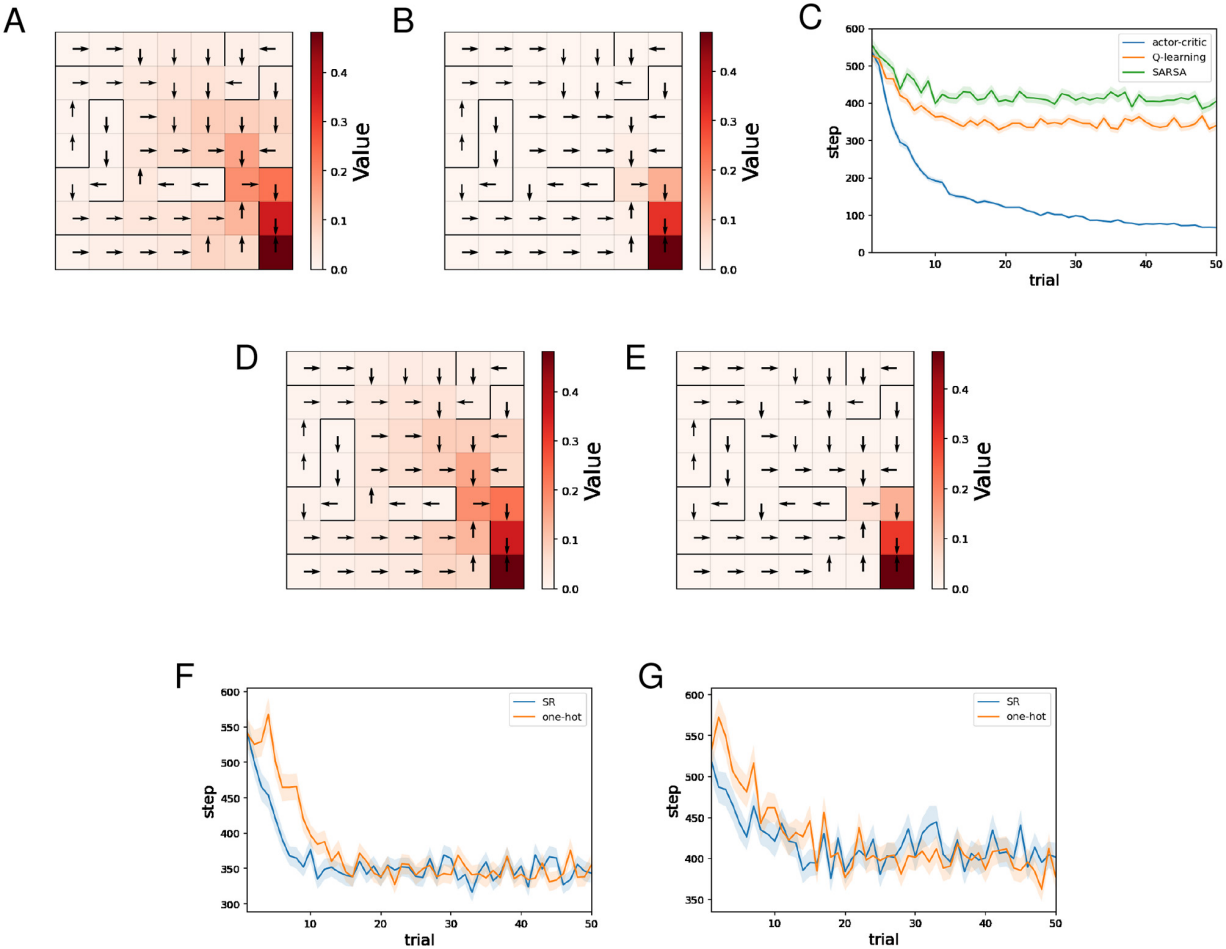
The results of the barrier maze task regarding Q-learning and SARSA. **(A, B)** Typical examples of the learned value function and optimal actions at the 50th trial of Q-learning **(A)**/SARSA **(B)** using the SR. **(C)** Performance comparison between the actor-critic method, Q-learning, and SARSA. **(D, E)** Typical examples of the learned value function and optimal actions at the 50th trial of Q-learning **(D)**/SARSA **(E)** using one-hot encoding. **(F)** Performance comparison between Q-learning using the SR and Q-learning using one-hot encoding. **(G)** Performance comparison between SARSA using the SR and Q-learning using one-hot encoding.

Figures 5A, B show typical examples of the learned value function and optimal actions for Q-learning and SARSA using the SR, respectively. As in the water maze task, we can see that learning of the value function proceeds faster for Q-learning than SARSA. The learned optimal actions are appropriate even in short paths with dead ends.

Figure 5C shows the mean and standard error of the number of steps for each trial while training sessions of 50 trials were performed 500 times, for different learning methods using the SR. As for AC, the SR is used both for the Critic and the Actor. As in the water maze task, we can see that learning by AC was significantly faster than Q-learning and SARSA, while Q-learning was slightly faster than SARSA.

Figures 5D, E show typical examples of the learned value function and optimal actions for Q-learning and SARSA using one-hot encoding, respectively. In both cases, there is no significant difference compared to when the SR is used.

Figure 5F shows the mean and standard error of the number of steps for each trial while training sessions of 50 trials were performed 500 times, for Q-learning with the SR and Q-learning with one-hot encoding. Learning by Q-learning with the SR was slightly faster than learning by Q-learning with one-hot encoding. Figure 5G shows the mean and standard error of the number of steps for each trial while training sessions of 50 trials were performed 500 times, for SARSA with the SR and SARSA with one-hot encoding. There is no significant difference in learning speed.

### 3.3 Policy revaluation task

**Figure 6:**
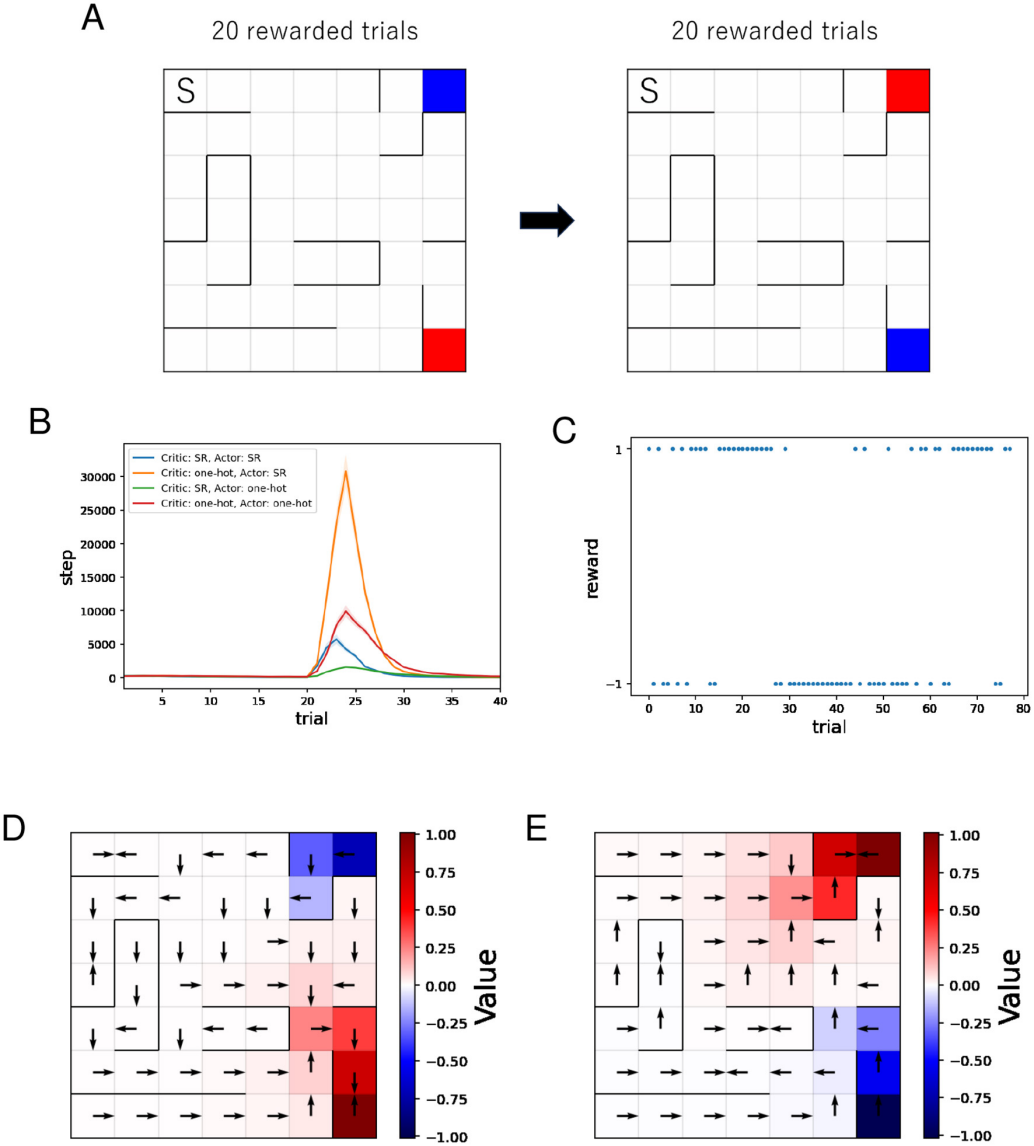
The results of the policy revaluation task. **(A)** The agent learns value and policy in each placement in order. The location marked “S” represents the starting point, the red location represents the reward location, and the blue location represents the punishment location. **(B)** Performance comparison between four different combinations of state representations. **(C–E)** Typical results when the SR is used for the Critic and one-hot encoding is used for the Actor. **(C)** History of rewards earned during the learning process. Negative rewards are punishment. **(D, E)** Learned value function and optimal actions before **(D)**/after **(E)** the reversal.

As previously described, in Figure 6A, the agent learns value and action in each placement in order. Then, Figure 6B shows the mean and standard error of the number of steps for each rewarded trial while training sessions of 40 rewarded trials were performed 500 times. Because we set an upper limit on the total number of steps per session during simulation, some runs end before reaching the reward location 20 times after reversing the reward location and the punishment location. Such runs are excluded from the mean and standard error. The number of such runs was 5 for “Critic: SR, Actor: SR,” 148 for “Critic: one-hot, Actor: SR,” 0 for “Critic: SR, Actor: one-hot,” 1 for “Critic: one-hot, Actor: one-hot.” Therefore, the actual difference between “Critic: one-hot, Actor: SR” and other combinations is larger than the plot indicates. The number of steps required to reach the reward location increases after the reversal because the new reward location is the original punishment location. As learning progresses, the number of steps required to reach it decreases. When the SR is used for the Critic and one-hot encoding is used for the Actor, the increase in steps after the reversal is smallest. The plots in Figure 6B indicate that using the SR for the critic suppresses the increase in steps after the reversal, while using the SR for the actor results in a larger increase in steps after the reversal.

We analyze the learning process when using the SR for the Critic and one-hot encoding for the Actor, which showed the smallest increase in steps after the reversal, from perspectives other than the number of steps.

Figure 6C shows a typical example of the history of rewards earned during learning when the SR is used for the Critic and one-hot encoding for the Actor. In this example, the reward and punishment positions are reversed around the 30th trial. Immediately after the reversal, the probability of reaching the punishment position is higher than the probability of reaching the reward position because the policy at that time is to move toward the punishment position (= the original reward position). We can see that the probability of reaching the punishment position is decreasing again after that.

Figures 6D, E show typical examples of the learned value function and optimal actions when the SR is used for the Critic and one-hot encoding for the Actor. It can be seen that after the reversal, the new value function is being learned. Unlike in the barrier maze task, the learned optimal actions lead to dead ends in some short paths. Since this occurs on paths relatively close to the punishment location, it is reasonable to consider that the cause is that the learning effect to move away from the punishment location outweighs the learning effect to move toward the reward location in some short paths.

## 4 Discussion

We constructed models in which the SR computed in the brain is used by the brain to make action choices, and performed reinforcement learning using these models. Our experiments revealed the difference between the effect of using the SR for the Critic and the effect of using the SR for the Actor in the actor-critic method.

The actor-critic method outperformed Q-learning and SARSA in our experiments. Furthermore, in our model with Q-learning and SARSA, the number of neurons required to represent states increases by a factor of the number of actions, resulting in high computational costs.

In our model with the actor-critic method, the preference for actions in one state is influenced by the learning of actions in other states, when the SR is used for the Actor. It was suggested through our experiments that this situation can have both positive and negative effects on behavioral learning. Such inter-state influence can occur not only when the SR is used as state representation or when the actor-critic method is used for learning. This may be the case in behavioral learning in animals.

In previous studies on actor-critic methods with neural network models (Barto et al., 1983; Barto, 1995), the value function and action preference are calculated by applying weights to the input. In short, the input is used as ***x*** in Section 2.3. Thus, the same ***x*** is used for the Critic and the Actor, and no experiments are conducted using different ***x***. In our experiment, when using the SR for the Critic and one-hot encoding for the Actor, the SR and one-hot encoding complemented each other. The use of different state representations for the Critic and the Actor corresponds to the use of representations or activities in different regions of the animal brain for the evaluation of value and decision-making. It is possible that such use benefits behavioral learning in animals.

The model to calculate the SR (Fang et al., 2023) adopted in this study is supposed to be implemented in the hippocampus. Actor-critic methods are closely related to the basal ganglia (Barto, 1995; Houk et al., 1995), and it is hypothesized that the dorsolateral striatum corresponds to the Actor and the ventral striatum to the Critic (Takahashi et al., 2008). From this perspective, our model can be viewed as a model in which the basal ganglia perform value computation and action selection using the SR computed in the hippocampus. More specifically, the RNN part can be interpreted as a model of the hippocampus, and the neuron that represents the value function and the neurons that represent action preference can be interpreted as a model of the striatum.

The striatum is roughly divided into dorsal and ventral parts. The dorsal striatum is further divided into the dorsolateral striatum (or the putamen nucleus in humans) and dorsomedial striatum (or the caudate nucleus in humans), and the ventral striatum contains the nucleus accumbens. Those striatal subdivisions are connected to different cortical and subcortical structures, forming limbic (accumbal), associative (dorsomedial striatal) and sensorimotor (dorsolateral striatal) loops, respectively. In this study, we found that the combination that uses the SR for the Critic and one-hot encoding for the Actor has advantages in terms of learning accurate policies and adaptation to environmental changes. According to the hypothesis that the ventral striatum corresponds to the Critic and the dorsolateral striatum corresponds to the Actor, this combination corresponds to the ventral striatum using the SR and the dorsolateral striatum using one-hot encoding. Combining the hypothesis that SR is computed in the hippocampus (Stachenfeld et al., 2017) with the fact that the ventral striatum (particularly the nucleus accumbens) is in the limbic loop as mentioned above and thus receives projections from the limbic system including the hippocampus, it is indeed possible that the ventral striatum uses the SR. On the other hand, one-hot encoding is a state representation that corresponds more directly to the states than the SR. Therefore, it is suggestive that the dorsolateral striatum receives sensorimotor-related information rather than associative information. By the way, the ventral striatum is critical for goal-directed behaviors, while the dorsolateral striatum is critical for habitual behaviors (Burton et al., 2023). This is also consistent with the use of the SR by the ventral striatum and one-hot encoding by the dorsolateral striatum, because it is reasonable to use structured state representations such as the SR for goal-directed learning and simple state representations such as one-hot encoding for habitual learning.

## Data Availability

The codes for simulations are available at GitHub (https://github.com/tkyktrm/frontiers).
